# Cell morphologies in the nervous system: Glia steal the limelight

**DOI:** 10.1002/cne.25429

**Published:** 2022-10-31

**Authors:** Giorgio A. Ascoli

**Affiliations:** ^1^ Center for Neural Informatics, Structures, & Plasticity (CN3), Bioengineering Department, and Neuroscience Program George Mason University Fairfax Virginia USA

## Abstract

Neurons and glia have distinct yet interactive functions but are both characterized by branching morphology. Dendritic trees have been digitally traced for over 40 years in many animal species, anatomical regions, and neuron types. Recently, long‐range axons also are being reconstructed throughout the brain of many organisms from invertebrates to primates. In contrast, less attention has been paid until lately to glial morphology. Thus, although glia and neurons are similarly abundant in the nervous systems of humans and most animal models, glia have traditionally been much less represented than neurons in morphological reconstruction repositories such as NeuroMorpho.Org. This is rapidly changing with the advent of high‐throughput glia tracing. NeuroMorpho.Org introduced glial cells in 2017 and today they constitute nearly a third of the database content. It took NeuroMorpho.Org 10 years to collect the first 40,000 neurons and now that amount of data can be produced in a single publication. This not only demonstrates the spectacular technological progress in data production, but also demands a corresponding advancement in informatics processing. At the same time, these publicly available data also open new opportunities for quantitative analysis and computational modeling to identify universal or cell‐type‐specific design principles in the cellular architecture of nervous systems. As a first application, we demonstrated that supervised machine learning of tree geometry classifies neurons and glia with practically perfect accuracy. Furthermore, we discovered a new morphometric biomarker capable of robustly separating these cell classes across multiple species, brain regions, and experimental preparations, with only sparse sampling of branch measurements.

## Graphical abstract

Diversity of digitally reconstructed glia (astrocytes, oligodendrocytes, microglia, and other types) freely available for download and reuse at NeuroMorpho.Org in terms of experimental provenance, animal species, anatomical region, cell type, tracing platform, and more.

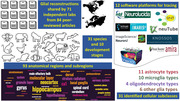

The nervous systems of almost all animals consist of two distinct, yet functionally interactive, cellular families: neurons and glia. Both of these constituents are characterized by branching morphologies. The trees stemming from glial cell bodies, referred to as processes, serve a variety of functions depending on the cell type, including myelination in oligodendrocytes, immune reactivity in microglia, and metabolic regulation in astrocytes. Most neurons emanate from their somas separate arbors specialized in signaling input (dendrites) and output (axons). Although certain neurons can receive stimuli directly from the external environment (sensory receptors), the vast majority of information transmission in the brain is mediated by directional synaptic contacts between axons and dendrites. Dendrites are endowed with considerable biophysical complexity and plasticity, making them prime effectors of sophisticated physiological mechanisms of neural computation, such as data integration, storage, and retrieval. Axons are responsible for connecting neurons both in local circuits (interneurons) and across the whole extent of nervous systems (projection neurons), providing the means for fast and reliable communication.

Dendritic trees have been three‐dimensionally traced from light and electron microscopy into computer‐readable digital formats for over four decades in many animal species, anatomical regions, and neuron types (Halavi et al., 2012). On the one hand, such intense data collection was fueled by the considerable theoretical interest of the scientific community in dendrites. On the other, it was practically made possible by the tractable spatial span of relatively contained arbors sparsely stained by traditional silver impregnation, intracellular injections, or, more recently, genetically encoded fluorescent proteins. In the last few years, thanks to parallel technological breakthroughs in labeling, imaging, and informatics, long‐range axons also are being reconstructed throughout the brain of many organisms from invertebrates (Shih et al., 2020) to primates (Zhou et al., 2022), which is beginning to reveal the cellular architecture of regional interactions.

Digitally tracing neural morphology is a considerably time‐consuming process, but the resulting reconstruction files are very information rich and can be utilized in many scientific applications beyond the specific research purpose they were originally intended for. In order to facilitate open community sharing and re‐use of these data, in 2006 we launched NeuroMorpho.Org, a public repository of reconstructed dendritic and axonal arbors from any animal species, developmental stage, anatomical region, cell type, preparation protocol, imaging method, and tracing software (Ascoli, 2006). The free availability of such diverse datasets spawned the emergence of a synergistic ecosystem of tools, models, and applications to visualize, analyze, and improve the acquisition of digital reconstructions of neural morphologies (Parekh & Ascoli, 2015). In particular, the development of new algorithms for automated tracing triggered a virtuous circle of increased data production, greater sharing, and multiplied opportunities for meta‐analyses and big science exploratory mining (Peng et al., 2015).

Compared to neuronal axons and dendrites, lesser attention has been paid until lately to glial morphology. As a consequence, although glia and neurons are similarly abundant in the nervous systems of humans and most animal models (Herculano‐Houzel et al., 2015), glia have traditionally been much less represented than neurons in the morphometric literature and in NeuroMorpho.Org. This is radically changing with the advent of high‐throughput glia tracing (Figure 1). Glial cells were introduced in NeuroMorpho.Org in 2017 (Akram et al., 2018) and today microglia, astrocytes, and oligodendrocytes constitute nearly a third of the database content (Figure 1a). It took NeuroMorpho.Org 10 years to collect the first 40,000 neurons (Ascoli et al., 2017) and now that amount of data can be produced in a single publication (Figure 1b). Such a dynamic playing field not only demonstrates the spectacular technological progress in data production, but also opens new opportunities for quantitative analysis and computational modeling.

**FIGURE 1:**
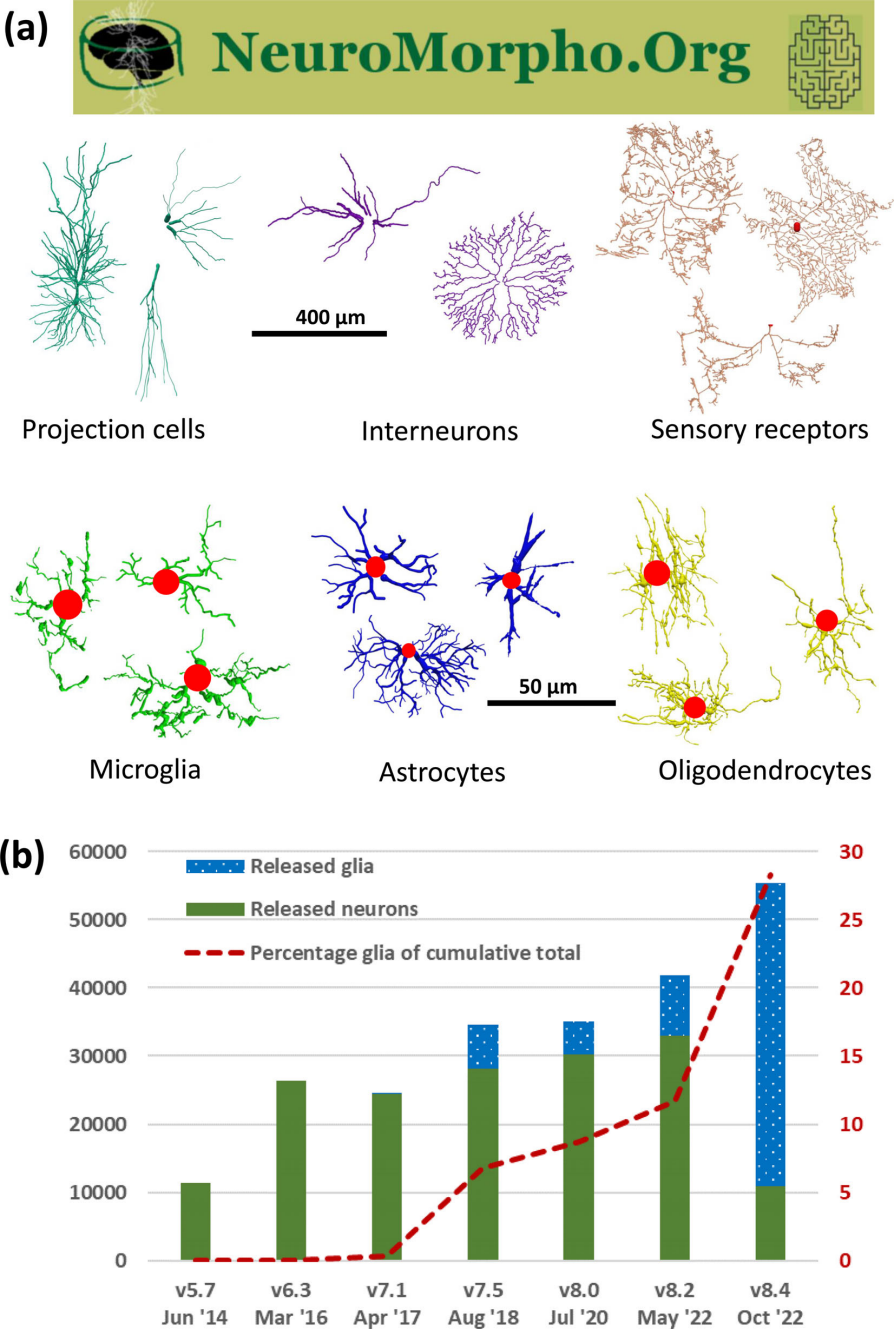
Digital reconstructions and public sharing of neural morphology in NeuroMorpho.Org. (a) Examples of reconstructed neuronal dendrites (top series) and glial processes (bottom series) from projection cells (left cell: Dalrymple‐Alford et al., 2015; top cell: Borrie et al., 2014; bottom cell: Yamada et al., 2016), interneurons (left cell: Deng et al., 2016; right cell: Bloomfield & Miller, 1986), sensory receptors (top cells: Sulkowski et al., 2011; bottom cell: Clark et al., 2018), microglia (left and bottom cells: Becchi et al., 2017; top cell: Ohgomori et al., 2016), astrocytes (top and bottom cells: Diniz et al., 2016; right cell: Canchi et al., 2017), and oligodendrocytes (top cell: Toth et al., 2021; bottom and right cells: Fannon et al., 2015). (b) Newly released neuronal and glial tracings in NeuroMorpho.Org

Pioneering advances in digital reconstructions of glia morphology gained momentum with the quantitative profiling of microglia processes in rat motor cortex (Megjhani et al., 2015) followed by an investigation of basal ganglia neuroinflammation in a Parkinson's disease model (Krashia et al., 2019). Astrocytes were also traced in the amygdala of rats (Wahis et al., 2021) as well as in cross‐species comparisons including neocortex of 21 distinct mammals from bat, anteater, and sloth to panda, bonobo, and human (Falcone et al., 2019) and hippocampus of migratory shorebirds (Mendes de Lima et al., 2019). Oligodendrocyte precursor cells were reconstructed as well during Central Nervous System ontogenesis in the mouse (Lorenzati et al., 2021).

In this context, we view the recent work by a team of investigators led by Sandra Siegert (Colombo et al., 2022) as a substantial leap forward. Siegert and coauthors digitally reconstructed an extraordinary number of microglia from the mouse brain across anatomical regions, sexes, developmental stages, and physio‐pathological states. The sheer amount of data publicly released with their work exceeds the average sample size in peer reviewed publications by two‐and‐a‐half orders of magnitude; it is more than 2.6‐fold larger than the previously largest single‐lab release (Nanda et al., 2015); and it nearly triples the total count of publicly available glial reconstructions from all animals, neural systems, and research groups combined. To tackle this exceptionally large dataset, the authors of this study devised an analytic workflow integrating topological descriptors (as in Li et al., 2017) and data science (as in Polavaram et al., 2014) to complement their systematic experimental pipeline. The resulting comprehensive quantification of microglia morphology identified a dimorphic and brain region‐dependent alteration of branching patterns throughout postnatal development and disease models. Besides their intrinsic significance, these findings also provide an important starting point for multiple new lines of investigation including environmental manipulations (such as diet, exercise, stress, sleep, and injury) and pharmacological interventions. Admirably, these investigators also chose to distribute the source code for their statistical toolset, which combines the previously introduced persistence homology barcoding (Kanari et al., 2018) together with bootstrapping, dimensionality reduction, and display transform. These same techniques and associated software could be widely applied by many independent researchers to similarly address other related questions in neurobiology (Gleeson et al., 2017).

At the current rate of data production, the number of digitally reconstructed glia freely downloadable from NeuroMorpho.Org will likely pass 100,000 by 2026. What new research prospects can the availability of such large‐scale scientific collections open? As a first application, we have recently demonstrated that supervised machine learning of tree geometry classifies neurons and glia with practically perfect accuracy across multiple species, brain regions, and experimental preparations (Akram et al., 2022). This study led to the discovery of a new morphometric biomarker, the average branch Euclidean length (ABEL), capable of robustly separating these cell classes with only sparse sampling of branch measurements. This study also illustrates a broader point: while small datasets generated by single labs are adequate to test specific hypotheses with suitably designed experiments, much larger datasets spanning a diversity of conditions (Figure 2) additionally enable the execution of brute‐force exploratory analyses. We thus predict an increasing trend in more frequent application of machine learning to the investigation of glia morphology (Labate & Kayasandik, 2023).

**FIGURE 2:**
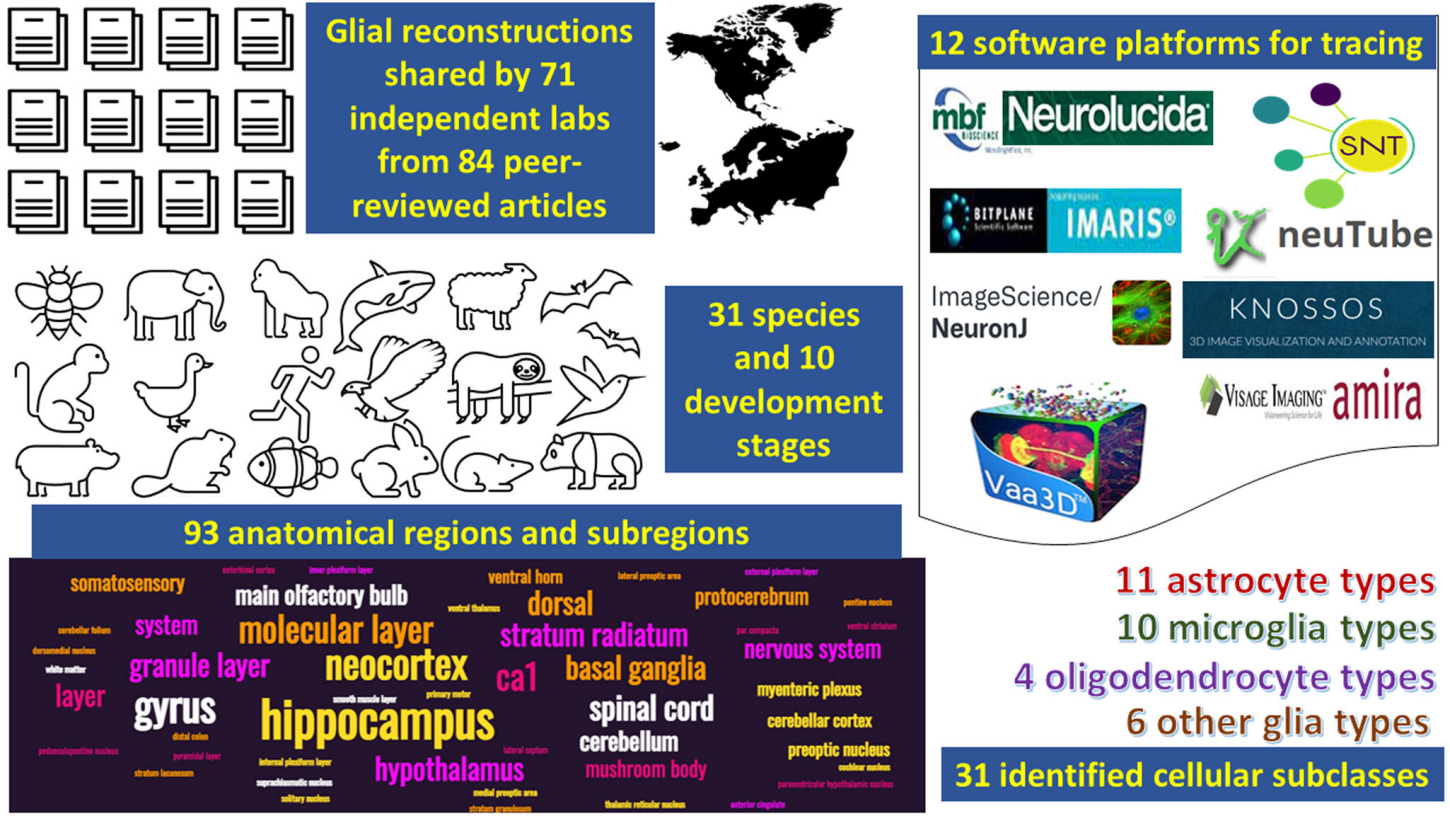
Diversity of reconstructed glia freely available for download and reuse at NeuroMorpho.Org in terms of experimental provenance, animal species, anatomical region, cell type, tracing platform, and more

My personal perspective on the potential for future high‐impact applications is inspired by the many success stories of scientific breakthroughs obtained by secondary usage of shared data (Anderson et al., 2021). These can be coarsely grouped by research theme, and are briefly exemplified below, as meta‐analyses and classification; physical modeling; potential connectivity analysis; structure‐activity‐function relationship; and morphological simulations. Classifying cells is important to elucidate their systems‐level functional roles. Neurons have historically been divided into types by the shape of their arbors (Hamilton et al., 2012), but this approach has been relatively less explored for glia. Efficient metrics and high‐throughput tools for large‐scale comparisons (Bijari et al., 2021; Ljungquist et al., 2022) of heterogeneous glial populations may soon allow to discover the individual morphological signatures of microglia, astrocytes, oligodendrocytes, and other glial types.

Morphological reconstructions provide precise measurements of the physical placement and spatial occupancy of neural arbors. This information has been leveraged to correlate neurite orientation with macroscopic white fiber tracts (Scholtens et al., 2022), to optimize insertion of intracranial electrodes (Paulk et al., 2022), to compute potential synaptic connectivity in a circuit (Tecuatl et al., 2021), and to estimate neurological radiation damage from cancer therapy or space travel (Cucinotta & Cacao, 2019). Extending these approaches to glial might allow one to predict patterns of myelination based on branching orientation in oligodendrocyte (or Schwann cells in the peripheral nervous system) relative to nearby neuronal axons. Investigating the coembedding of neurons, astrocytes, and microvasculature could lead to a deeper mechanistic understanding of the brain‐blood barrier (e.g., Zisis et al., 2021). Quantifying the distribution of activated microglia with respect to a traumatic epicenter may facilitate testing hypotheses regarding their inflammatory response.

Computational models using cable and Hodgkin‐Huxley equations have long contributed to elucidating the biophysical bases of neuronal electrophysiology (e.g., Lazarewicz et al., 2002). Glia are not excitable cells, but they are nonetheless known to affect information processing by gating neurotransmitter reuptake and by modulating synaptic plasticity. It should be especially revealing to adapt pharmacologically relevant neuronal models of intracellular calcium dynamics (e.g., Ferrante et al., 2008) to the branching architecture and biochemical cascades of astrocytes (Verkhratsky & Nedergaard, 2016). A different kind of computer simulations entails the digital synthesis of virtual neuronal morphologies, which can lead to the quantitative confirmation of developmental phenomena such as chemotropic growth (Samsonovich & Ascoli, 2003). Do the robust geometric properties discovered for neuronal axons and dendrites (Snider et al., 2010) also apply to glia? The answer will indicate whether the cellular architecture of nervous systems follows universal or cell‐type‐specific design principles.

Neurons have long eclipsed glia in commanding the center stage of neuromorphological research. The once overlooked glial processes, now emerging from the shadow, are ready to fuel a renaissance of discovery.

## CONFLICT OF INTEREST

The author declares no conflict of interests.

### PEER REVIEW

The peer review history for this article is available at https://publons.com/publon/10.1002/cne.25429.

## Data Availability

The data that support the findings of this study are available in NeuroMorpho.Org at https://NeuroMorpho.Org. These data were derived from the following resources available in the public domain: ‐ NeuroMorpho.Org, https://NeuroMorpho.Org.
